# Schlieren visualization of ultrasonic standing waves in mm-sized chambers for ultrasonic particle manipulation

**DOI:** 10.1186/1477-3155-11-21

**Published:** 2013-06-28

**Authors:** Dirk Möller, Nicolas Degen, Jurg Dual

**Affiliations:** 1 Department of Mechanical and Process Engineering, Institute of Mechanical Systems, ETH Zurich, Tannenstrasse 3, Zurich, Switzerland

**Keywords:** Schlieren visualization, Schlieren setup, Ultrasonic particle manipulation, Pressure field

## Abstract

**Background:**

For the design and characterization of ultrasonic particle manipulation devices the pressure field in the fluid cavity is of great interest. The schlieren method provides an optical tool for the visualization of such pressure fields. Due to its purely optical nature this experimental method has got some unique advantages compared to methods like particle tracking.

**Results:**

A vertical schlieren setup and an investigation with the same of a mm-sized chamber used to agglomerate particles are presented here. The schlieren images show a two-dimensional representation of the whole pressure distribution recorded simultaneously with a good resolution in time. The gained description of the pressure field is shown to be in agreement with a numerical simulation. Thermal effects as well as streaming effects are shown.

**Conclusions:**

The results show the great potential of schlieren visualization to investigate ultrasonic particle manipulation devices. Visualized are pressure fields, acoustic streaming, temperature effects and effects caused by fluid volumes of different density.

## Background

The schlieren method is a proven tool for the experimental characterization of optically transparent liquids and for the visualization of ultrasonic fields [1]. This method makes visible spatial variations in the refractive index of the liquid. Thus any quantity related to the refractive index can be visualized, these are e.g. temperature variations or density variations caused by fluid flow or by acoustic waves. The method is similar to shadowgraphy and first known reports of a schlieren setup are from R. Hooke in the year 1665 for the visualization of thermal disturbances in air. The term schlieren goes back to A. Töpler who reported the first advanced schlieren setup in the year 1864. A more modern account on the schlieren technique in air is given by G.S. Settles [2]. Reports suggest a large variety of different setups based on mirrors and transparent lenses including bidirectional setups with a mirror as background [3]. Specialized or similar methods are e.g. the background oriented schlieren method [4], the colour schlieren method [2] or phase contrast and interferometry methods such as the differential interference contrast method.

Acoustic radiation forces [5] are exerted on particles in an ultrasonic standing pressure field. Particles which are denser and stiffer than the surrounding medium are driven towards the pressure nodes. These forces can be used for the transport and agglomeration of cells or micrometer-sized particles in suspension with a major area of application in life-sciences and biochemistry, such as drug screening or purification of nucleic acid solutions for sensitive molecular analysis [6,7]. For ultrasonic particle manipulation devices, particle tracking [8] is also a very strong tool to characterize the pressure field or to visualize fluid flow. While ultimately both methods can be combined, the schlieren method offers some unique advantages. In contrast to particle tracking, its timescales are not limited by drag forces so it is much faster and, with the proper equipment, even able to measure traveling waves. In addition it is a non intrusive method, that is no seeding particles are required which might influence the fluid properties or the acoustic field. Other advantages of an optical method are that the complete area of interest can be imaged simultaneously and constantly even for fast frequency changes in real time while resolving complete pressure waves of a standing wave field. The resolution and frequencies of the ultrasonic field which can be imaged are mainly limited by the optical wavelength and the resolution of the optical components used. Reports show schlieren images of ultrasound in water of over 100 MHz [9]. The structure containing the fluid, like any obstacle in the optical light path, causes interference fringes which can be a limiting factor for small devices or for imaging features close to a wall. The same problem can arise when using the schlieren method in combination with particles.

## Method and setup

**Figure 1:**
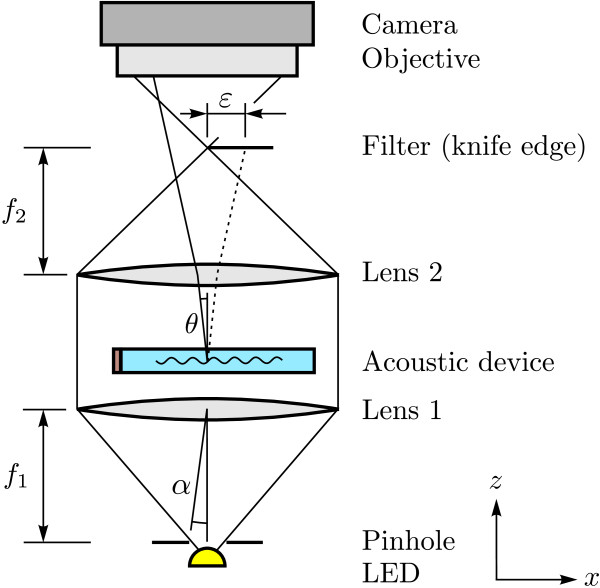
Schematic of vertical schlieren setup with device in the object plane and a knife edge filter.

The setup presented here is a conventional (Töpler) three lens schlieren system which has been mounted vertically as shown in Figure 1. The single components can easily be exchanged. For a large ratio of focal length *f*_1_ to pinhole diameter *p* the relation *α*∝*p*/*f*_1_ holds, where *α* is the beam deviation angle at the collimation lens L1 which should be as small as possible. For *p* there is a lower limit in the order of the Airy disc and Rayleigh criterion $p=2\sqrt{2f_{1}\lambda }$, where *λ* is the optical wavelength. The diffraction angle *θ* is given by sin*θ* = *λ*/*Λ* for the first intensity peak, where *λ* is the acoustic wavelength. The diffracted beam separation distance *ε* = *θf*_2_ is a measure for the sensitivity of the schlieren setup. For the work presented here the two field lenses (L1, L2) have a lens diameter of 50 mm where lens L1 is a planoconvex lens with a focal length of 250 mm and lens L2 is an achromatic lens doublet with a focal length of 400 mm which is also corrected for spherical aberration and coma. As light source a modified high power white LED with a 300 *μ*m pinhole has been used. Since the acoustic devices are operated in quasi stationary mode relatively long camera exposure times can be used as compared to imaging of traveling waves. This eases the requirements for the light source and camera in terms of brightness. In the cut-off plane either a knife edge filter which allows to emphasize directional effects dependent on its rotational position or a dark field filter has been used. The camera objective is a Minolta 135 mm and the camera is a Lynx IPX-2M30-G resulting in a spatial resolution in the order of 20 *μ*m /pixel. The focal plane of the objective is at the position of the acoustic device resulting in an image where the spacing of the interference fringes is equal to the ultrasonic wavelength multiplied by the lateral magnification of real images [1].

With a device that uses continuous frequency sweeping in combination with standing pressure fields, particles can be moved over larger distances using acoustic radiation forces [10]. In macro scale chambers even a small change in frequency can change the pressure field significantly and thermal convection as well as acoustic streaming can be of significance, thus schlieren imaging is particularly suited to investigate such a device experimentally. The devices [11] investigated have a square fluid volume with a base of 21 x 21 mm and a height of 3 mm which is the same as the assumed acoustic beam diameter *L*. They are operated in the lower MHz frequency range. Assuming the optical axis to be parallel to the ultrasonic wave front and an optical wavelength *λ* of at least 700 nm the Klein-Cook parameter *Q* = 2 *πλL*/*Λ*^2^ ≪ 1 and thus Raman-Nath diffraction [12] is expected. In other words, the acoustic beam diameter *L* or length that the optical waves cross the acoustic waves, is sufficiently small for the effect of multiple diffraction to be neglected. The devices are excited with a 1 mm thick piezo electric element, Pz26, which is either directly glued to one of the chamber walls or first glued to an aluminium waveguide and then clamped to the device. The excitation signal is a frequency sweep of 1.5 MHz to 2.5 MHz modulated with a saw-tooth frequency of 0.05 Hz. This type of excitation allows to move particles or cells from one side of the chamber to another or back by reversing the frequency range. The devices are built up with three layers, a PMMA frame with 0.5 mm thick walls and a planar top and bottom cover which are either 250 *μ*m PMMA foils or 0.5 mm glass slides. The glass is introduced to minimize disturbances in the optical path. A comparison of both cover materials did not show significant differences in the qualitative image of the acoustics.

## Results and discussion

**Figure 2:**
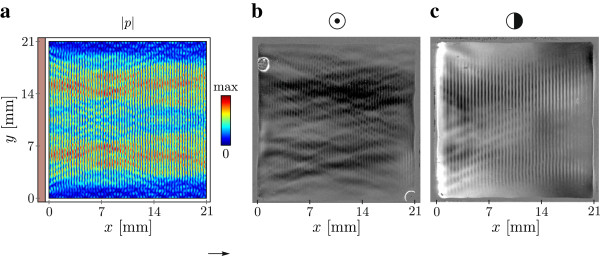
**Top view on acoustic device with numerical simulation and schlieren images. a)** Absolute pressure field |p| obtained with a FEM simulation as colour graph at 2.105 MHz, 0.5 mm PMMA frame (white) and piezo element (brown). **b)** Dark field filter grain extract schlieren image at 2.105 MHz. **c)***x*-direction knife edge filter grain extract schlieren image at 1.650 MHz. In the schlieren images the darkest shade is a pressure node for image areas with a local change of gray shades.

Top views of the device are shown in Figure 2, where the *z*-axis is along the optical axis of the schlieren setup. Figure 2 a) shows a FEM simulation performed with COMSOL Multiphysics 4.2a in 2D of the device, where the transducer is depicted in brown and as colour graph the absolute pressure field |p| is shown at a frequency of 2.105 MHz. The pressure field shows an inhomogeneous distribution with areas of multiple wavelengths which have a higher maximum pressure. These areas can change their position along the *y*-axis significantly with a relatively small frequency change in the order of 1 kHz. This is an example that would be difficult to see with particle tracking as these changes are often too fast and or the amplitudes, and with it the primary acoustic radiation forces are too low. For many frequencies the simulation also shows low pressure amplitudes close to the two boundaries along *y* = 0 mm and *y* = 21 mm.

The schlieren images are shown as grain extract images. For images with a linear intensity range from 0 (black) to 1 (white) a grain extract image *G* is obtained with *G* = min(1, max(0,*B*−*F*+0.5)) where *B* is the background image and *F* the front image. The front image is the schlieren image of interest. The resulting grain extract images have a pressure node where the local intensity field is the darkest, for both knife edge filter and dark field filter. Areas within the fluid chamber without a standing pressure wave present, or with one of low amplitude show no local variation in the schlieren image. Figure 2 b) and c) show grain extract images where *B* is a schlieren image of the device without ultrasound and *F* a snap-shot image during sweeping at 2.105 MHz and 1.650 MHz obtained with the schlieren setup. For the image in b) a dark field filter has been used, revealing both the standing pressure field as well as changes in *x*- and *y*-direction. These changes appear to be in line with the numerical simulation, both in shape and response to small frequency changes. The schlieren image in c) is obtained with a knife edge filter in *x*-direction. The visualized nodal pressure planes show to be almost parallel with some disturbances around the four boundaries. The bright white stripe at the boundary along *x* = 0 mm close to the piezo element, as well as the other disturbances close to it are due to thermal gradients and convection caused by heat dissipation of the transducer. For prolonged experiments with high driving voltages and without cooling these thermal effects appear very clearly on the schlieren images.

The rather weakly developed schlieren image at the side boundaries (*y* = 0 mm and *y* = 21 mm) are in good agreement with the results from the numerical simulation as well as with the experiments with particles. They all indicate that close to these boundaries the pressure amplitudes are quite low. The schlieren images given here are from devices with 1 mm openings in two opposing corners (*x*,*y*) = (0,21) and (21,0) which will also have an effect on the pressure on the boundary. However experiments with closed cover slides as well as the numerical simulation which are both without these openings, indicate that the low pressure is only partly due to these openings. Some disturbances can also be caused by convection in the air, even though they should be small in a vertical setup where the airflow of rising air is parallel to the light path.

**Figure 3:**
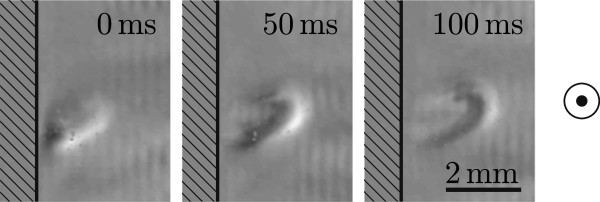
Three consecutive grain extract schlieren images of a wave front caused by acoustic streaming.

Figure 3 shows a wave front caused by acoustic streaming. The streaming itself is caused by some disturbance like a small air bubble at the boundary towards the transducer. With a changing excitation frequency the resonance frequency of the disturbance is only met for a small time window. The background image *B* used for the three shown grain extract images is an image right before the streaming burst starts. Like this any static part or low velocity change as e.g. the bubble will be suppressed, emphasizing high velocity changes like caused by fluid flow. The time passed between two images is 50 ms.

**Figure 4:**
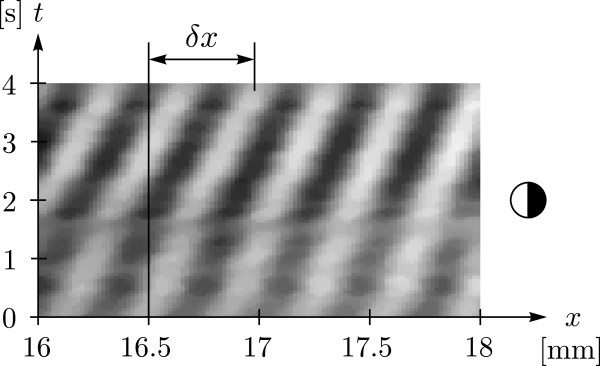
**Schlieren image showing the pressure distribution of a standing pressure wave over time.***δx* indicates the distance that a particle trapped in a pressure node would travel at this position with a frequency sweep from 2.1 MHz to 2.3 MHz which corresponds to a time span of 4 s.

For the transport of particles the evolution in time of the pressure field is of interest. As an example a row of pixels from grain extract schlieren images over a time period of 4 s is shown in Figure 4 for the same experiment as shown in Figure 2 c). The pixel row is located along a line reaching from *x* = 16 mm to *x* = 18 mm at *y* = 4 mm in the reference frame given in Figure 2. This time period is equivalent to a frequency range of 0.2 MHz and assuming an idealized 1D resonator, this equals to six fluid resonances within this range. The center frequency is around 2.2 MHz. The qualitative nature of the schlieren image shows that in the given region the maximal pressure amplitude remains almost the same over several fluid resonances except for some disturbances between 0 s and 2 s. For this frequency range particles trapped in a pressure node would travel by the distance *δx* if started at *x* = 16.5 mm. For a starting point of particles closer to the transducer, that is with decreasing *x*, the distance *δx* increases. However the given uniformity of the pressure amplitude over time and periodicity over space of the pixel area shown here is not given for the whole fluid chamber within the same frequency range. Therefore a larger range spanning over 1 MHz is used.

**Figure 5:**
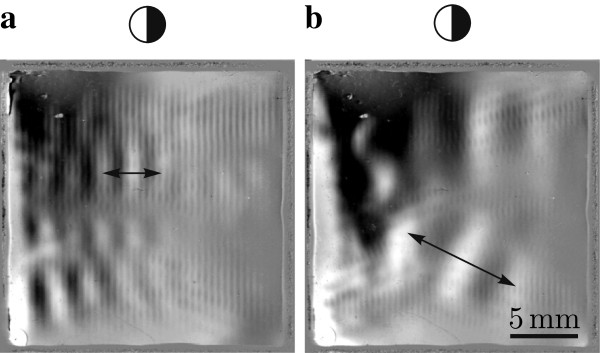
**Schlieren visualization of acoustic pressure field and lower harmonics.** Excitation frequencies are 1.50 MHz in **a**) and 1.71 MHz in **b**). The arrows indicate one wavelength.

With liquids of different density in the chamber even further phenomena can be visualized. One example is shown in Figure 5 where two different lower harmonics are shown together with the pressure field. The wavelengths of the harmonics are indicated with arrows. Compared to the thermal effects presented with Figure 2 c) here the duration of the experiment is much longer resulting in larger volumes of water with different temperatures. Without acoustics the fluid volumes of different densities arrange according to gravity resulting in gradients along the optical axis which are not visible with the schlieren setup. With active acoustics the fluid volumes slowly rearrange with gradients in the *xy*-plane which can be visualized with the schlieren setup. The transient time for the change of these gradients is in the order of seconds. Structural vibrations in comparison have a significantly lower decay time, typically two or three orders of magnitude lower for the same frequency as these lower harmonics.

## Conclusion and outlook

Schlieren visualization has proven to be a strong and simple tool to visualize standing pressure waves in ultrasonic micro manipulation devices. Both local and large scale pressure distributions can be imaged. Streaming effects as well as thermal effects can be studied at the same time as the pressure field. The method appears to be particularly suited to image changes over time while keeping the full resolution of the field. The quality of the imaging setup has the potential to be improved in order to record with higher sensitivity and homogeneity which can be achieved by introducing colour filters and light blocking filters of different shape [13] or with a more sophisticated lens setup and light source.

## Competing interests

The authors declare that they have no competing interests.

## Authors’ contributions

DM designed the ultrasonic device, performed the experiments and simulations and wrote the manuscript. ND proposed, built and tested an initial schlieren setup. DM refined the schlieren setup. JD contributed to the design of the study, revised the manuscript and is responsible for the research group. All authors read and approved the final manuscript.
